# Bufalin enhances antitumor effect of paclitaxel on cervical tumorigenesis via inhibiting the integrin α2/β5/FAK signaling pathway

**DOI:** 10.18632/oncotarget.6840

**Published:** 2016-01-07

**Authors:** Fei Liu, Duo Tong, Haoran Li, Mingming Liu, Jiajia Li, Ziliang Wang, Xi Cheng

**Affiliations:** ^1^ Department of Gynecologic Oncology and Cancer Institute, Fudan University Shanghai Cancer Center, Shanghai, China; ^2^ Department of Medical Oncology, Fudan University Shanghai Cancer Center, Shanghai, China; ^3^ Department of Oncology, Shanghai Medical College, Fudan University, Shanghai, China

**Keywords:** cervical cancer, Bufalin, integrin α2/β5, FAK, paclitaxel

## Abstract

While Bufalin restrains primary tumorigenesis, the role of Bufalin in cervical cancer remains unclear. Here, we show that Bufalin can inhibit cervical cancer cell proliferation, block cell cycle in G2/M phase, induce cellular apoptosis and reduce cell metastasis through stimulation of p21^waf/cip1^, p27^cip/kip^, Bax and E-cadherin, and suppression of cyclin A, cyclin B1, CDK2, Bcl-2, Bcl-xl, MMP9 and SNAIL1. Further study suggests that Bufalin has no apparent damage to human normal cervical cells at the low concentration (<20nM), but increases the chemotherapeutic efficacy of paclitaxel. Mechanistic study reveals that Bufalin suppresses the integrin α2/FAK/AKT1/ GSK3β signaling. Finally, in vivo studies show that Bufalin blocks the Siha-induced xenograft tumor growth without detectable toxicity in the animals at the therapeutic doses, and the combination treatment of Bufalin and paclitaxel more efficiently inhibits xenograft tumor growth. Thus, Bufalin may be developed as a potential therapeutic agent to treat cervical cancer.

## INTRODUCTION

Cervical cancer is one of the leading gynecological malignancies worldwide and often diagnosed at an advanced clinical stage [1]. The 5 year survival rate of cervical cancer patients with stage III was 32.8%, but those with stage IV was only 7.1% [2, 3]. Chemotherapy and radiotherapy are the common therapeutic interventions for cervical cancer either as primary treatment, or adjuvant, or neoadjuvant therapy [4]. Now, drug-resistance has been a major challenge in the treatment of recurrent cervical cancer. Therefore, it is urgent to develop novel therapeutic agents for relapsing cervical cancer patients, especially for those resistant to paclitaxel- or platinum-based chemotherapy.

Bufalin is the major digoxin-like component of the traditional Chinese medicine Chansu [5, 6] and increasing evidences have demonstrated that Bufalin exerts its anticancer effect on various cancer types [7-10], such as lung, breast, liver and pancreatic cancer, by inhibiting cell proliferation, inducing cell apoptosis and cell cycle arrest, reversing multidrug resistance, and regulating the immune response. It has been reported that Bufalin synergized with akt inhibitor LY294002 to induce the apoptosis of lung cancer A549 cells [11], which was associated with the upregulation of Bax expression, the downregulation of Bcl-2 and livin expression, and the activation of Caspase-3. In addition, it was also found that gemcitabine combined with Bufalin enhanced the antitumor efficacy of gemcitabine in pancreatic cancer [12]. These studies suggested that Bufalin may be a potential regimen for combined chemotherapy. However, the effect of Bufalin and its mechanism on cervical cancer cells have not yet been thoroughly evaluated.

Integrins are heterodimeric cell adhesion receptors, which contain two different chains, α and β subunits [13]. Integrins mediate a wide variety of cell-cell and cell-matrix interactions that lead to cell migration, proliferation, differentiation and survival [14-16], by modulating the cell signaling pathways of transmembrane protein kinases such as Focal Adhesion Kinase (FAK) [17, 18]. Integrin/FAK signal pathway participates in the activation of akt and the inhibition of GSK3β, and then promotes tumorigenesis, cell metastasis and chemoresistance [19-21]. For instance, the inhibition of integrin α4 contributed to improving efficacy of current therapies of acute lymphoblastic leukemia (ALL) [22], which was provided chemoprotection by bone marrow. Recently, some studies showed that integrins have a close association with cervical tumorigenesis and metastasis [23-25]. Chatterjee et al. found that integrin α5β3 was one of the most important cell surface molecules regulating the invasive property of cervical tumor cells because of its associated gelatinase/MMP-2 activity [25]. Therefore, targeting the integrin/FAK signal pathway has emerged as one of the major therapeutic strategies in the treatment of cervical cancer.

In this study, we illustrated that Bufalin inhibited cell proliferation, induced cell cycle arrest and apoptosis, reducing cell metastasis *via* the suppression of intergrin α2β5/FAK signaling pathway. Meanwhile, we demonstrated that the combination of Bufalin and paclitaxel more efficiently inhibited cervical cancer cell proliferation *in vitro* and xenograft tumor growth *in vivo*, but not affected normal cervical cells.

## RESULTS

### Bufalin suppresses cell proliferation by inducing cell apoptosis and cell cycle arrest

**Figure 1:**
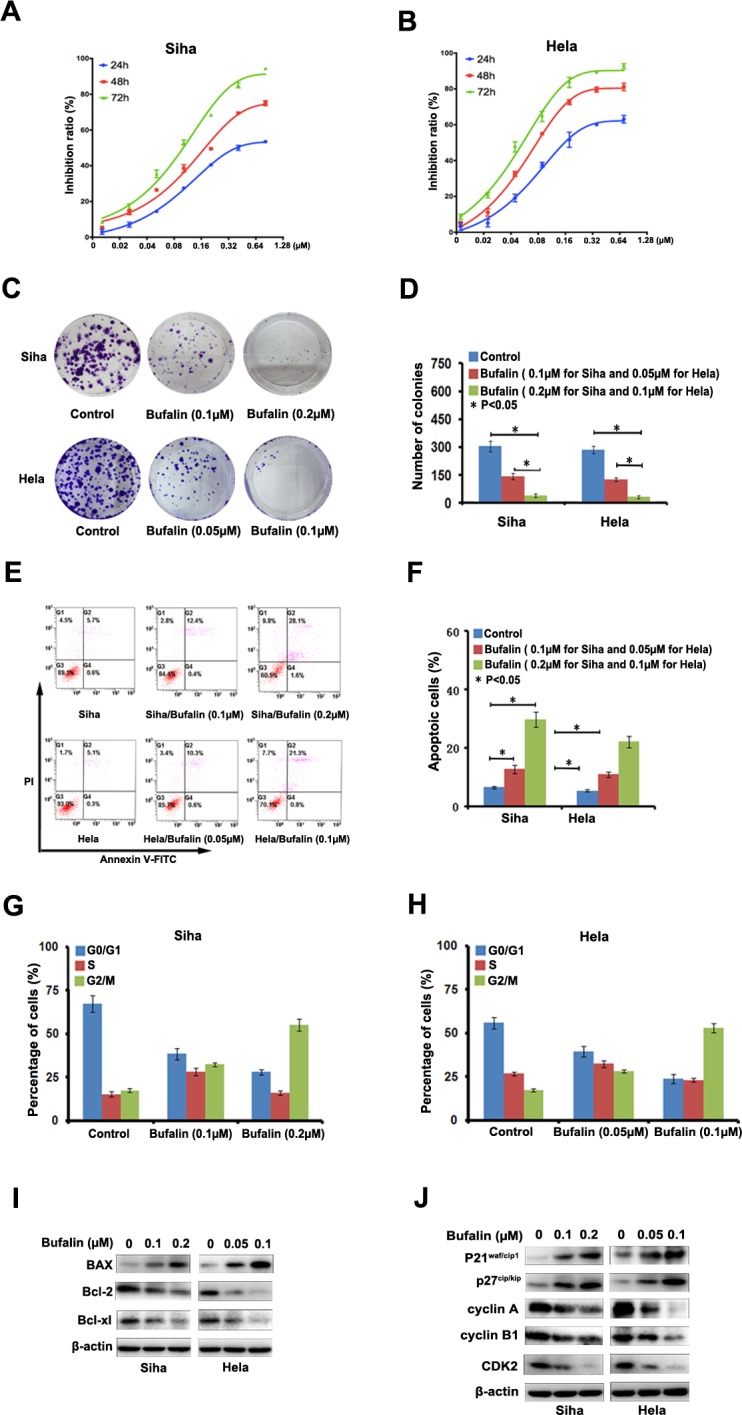
Bufalin inhibited cell proliferation and induces cell cycle arrest **A.**-**B.** Bufalin inhibited the proliferation of cervical cancer cell lines Siha and Hela. Cell viability determined by CCK8 assay. **C.** The apoptosis induced by Bufalin was detected by flow cytometry. **D.** Quantitative analysis of apoptotic cells (*P* < 0.05). Error bars = 95% CIs. **E.** Representative images of cell colonies after treatment with various concentrations of RY-2f for 48 h. **F.** Colony formation rate after treatment with RY-2f for 48 h. The experiments were repeated three times, and a representative experiment is shown. * *p* < 0.05. **G.**-**H.** Quantitative analysis of cell cycle distribution. Data from three independent experiments were analyzed (*p* < 0.05). Error bars = 95% CIs. **I.**-**J.** Immunoblotting analysis of apoptosis-associated and cell cycle regulatory proteins.

To examine the anticancer effects of Bufalin against cervical cancer, Siha and Hela cells were treated with Bufalin (0, 0.02, 0.04, 0.08, 0.16, 0.32, 0.64 and 1.28 μM) for 24, 48 and 72 h, and cell proliferation was assessed by CCK-8 assay. As shown in Figure 1A and 1B, the inhibition of the two cell lines increased with the increasing concentrations and duration of exposure time, which indicated that Bufalin suppressed cell proliferation in a time- and dose-dependent manner.

Next, we conducted colony formation assays to further determine Bufalin's inhibitory effects on cancer cell proliferation. The results clearly showed that the exposure to Bufalin reduced numbers and sizes of the colonies formed by the two tumor cell lines in a concentration-dependent-manner (Figure 1C). The numbers of colonies formed by cells treated with Bufalin or diluent were counted as shown in Figure 1D.

To determine the possible mechanism of the anti-cancer effects of Bufalin, we studied the induction of apoptosis after Bufalin treatment. After 24 hours of treatment with different concentrations of Bufalin and diluent, Siha and Hela cells were double stained by Annexin V and PI and subjected to flow cytometry to quantitatively analyze the apoptotic effects (Figure 1E). As illustrated in Figure 1F, the percentage of total apoptotic cells, including the early apoptotic portion (Annexin V positive) and the late apoptotic portion (Annexin V and PI positive), were dose-dependently increased with the raising concentrations of Bufalin in both cervical cancer cell lines. Besides, we also found that Bufalin treatment increased the pro-apoptotic protein Bax, but decreased the anti-apoptotic protein Bcl-2 and Bcl-xl in the both cancer cell lines (Figure 1I).

Previous studies have shown that bufatin could exert its anti-proliferative effect through blocking cell cycle. Thus, we also investigated the effect of bufatin on cell cycle regulation by flow cytometry analysis in the two cervical cancer cell lines, Sina and Hela. As shown in Figure 1G and 1H, We found that the cell population was decreased at the G0-G1 and S phase but increased at G2-M phases in both cell lines treated with Bufalin compared with in control cells. To explore the potential mechanism, we analyzed major proteins associated with cell cycle progression by Western blotting. The results in Figure 1J showed that p21 and P27, the essential negative regulators of cell cycle suppressor involved in the G1-S cell cycle transition, was dose-dependently increased in both cell lines after Bufalin treatment. The cyclinA/CDK2 complex plays a critical role in the transition of S/G2 phase. Our data showed that the levels of cyclin A and CDK2 were also reduced after Bufalin treatment, consistent with the reduction in S phase and G2/M arrest in flow cytometry analysis. Meanwhile, we found that Bufalin enhanced the expression of cyclin B1 (Figure 1J), indicating that cells was blocked at late stage of G2 phase and the accumulation of cyclin B1 finally triggered programmed cell death. Taken together, we provided strong evidence that Bufalin possess anti-cancer activities by inducing cell apoptosis and blocking cell cycle progression.

### Bufalin inhibits cervical cancer cell invasion and migration

**Figure 2:**
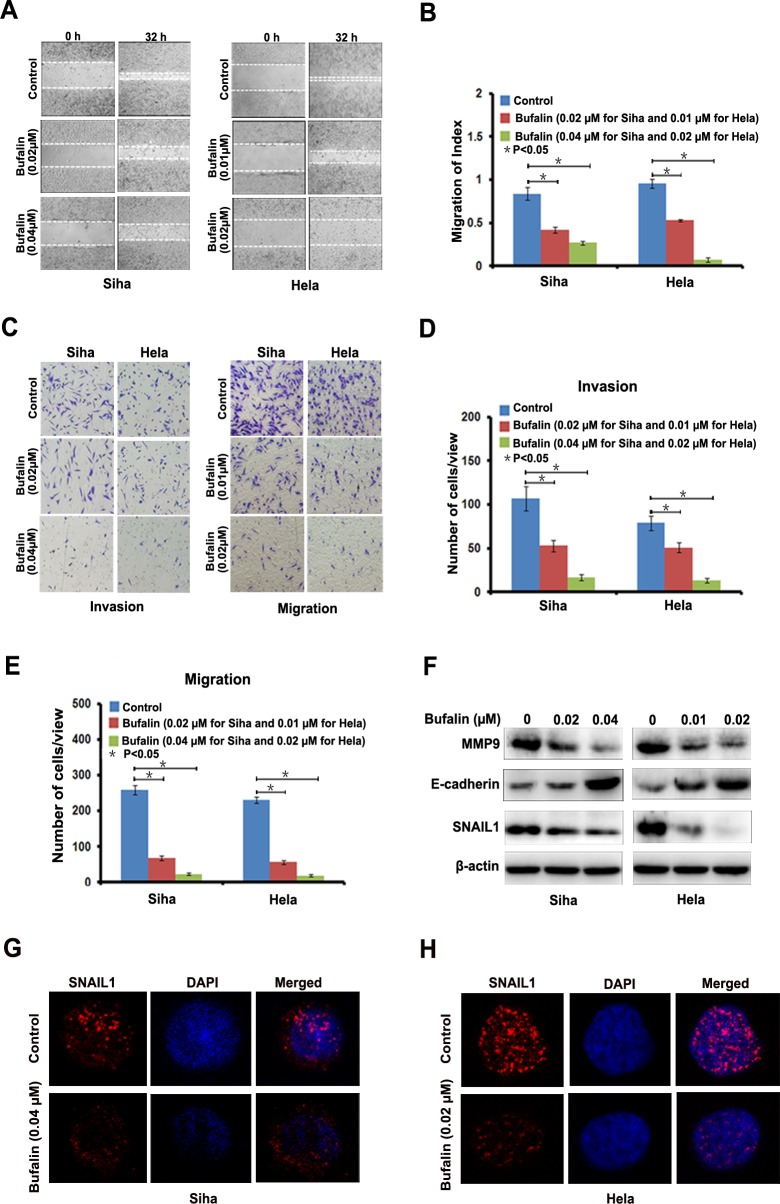
Bufalin suppressed cervical cancer cell migration and invasion by regulating EMT-associated proteins **A.** Detection of migration by scratching assay. B, Quantitative analysis of migration speed using migration index (*P* < 0.05). Error bars = 95% CIs. C, Detection of cell migration and invasion by using a high throughput screening multi-well insert 24-well two-chamber plates. D-E, Quantitative analysis of invaded cells (*P* < 0.05). Error bars = 95% CIs. F, Immunoblotting analysis of EMT-associated proteins. G-H, Representative images showing Bufalin inhibited the expression of Snail1 in cell nucleus with immunofluorescence staining (×1000). Blue dye DAPI indicates nucleus.

To evaluate the anti-metastatic potential of Bufalin, we performed scratch assay to detect cell migration speed and found that, compared with diluent, Bufalin dose-dependent decreased the migration speed of Siha and Hela cells (Figure 2A and 2B). We also found that Bufalin reduced the cells invaded through Matrigel and migrated through the membrane in the bottom chamber, which indicated that Bufalin could reduce the invasive and migratory abilities of the two cervical cancer cell lines (Figure 2C and 2D). Further, we determined the expression level of Epithelial-Mesenchymal Transition (EMT) -related proteins, including matrix metalloproteinase 9 (MMP 9), E-cadherin, and Snail1. Compared with control cells, after 24 hours exposure to Bufalin, the expression level of MMP 9 and Snail was dozes-dependent reduced, while E-cadherin was increased in both cell lines (Figure 2E). The results from immunofluorescence staining confirmed the down-regulation of snail1 in the cervical cells treated with Bufalin (Figure 2G and 2H). Based on these results, Bufalin appeared to halt cervical cancer cell invasion and migration possibly by simulating the expression of MMP9 and Snail1 and suppressing the expression of E-cadherin.

### Bufalin disrupts integrin α2/β5/FAK signal pathway

**Figure 3:**
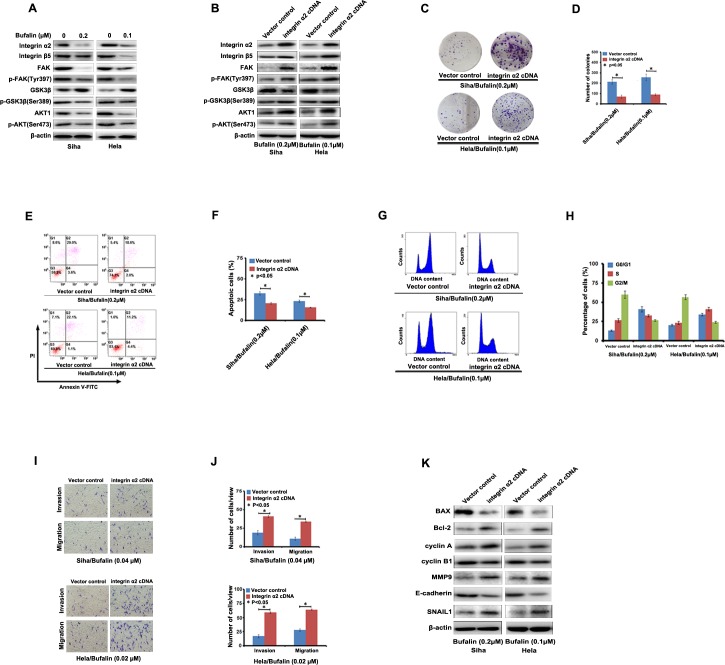
Integrin α2 rescued Bufalin-induced effect on cervical cancer cells **A.** Bufalin inhibited the expression levels of the members of integrin/FAK signaling detected by western blotting. **B.** Induction of integrin α2 cDNA rescued the Bufalin-induced effect on the expression of the members of integrin/FAK signaling detected by western blotting. **C.-F.** Induction of integrin α2 cDNA rescued the Bufalin-induced effect on apoptosis, cell cycle and mastastasis in cervical cancer cells.**G.** Induction of integrin α2 cDNA rescued the expression of apoptosis, cell cycle and mastastasis-associated proteins in Bufalin-treated cervical cancer cells.

In this study, we found that Bufalin interfered in integrin α2/β5/FAK signaling pathway. As shown in Figure 3A, the expression level of integrin α2 and β5 was suppressed in Siha and Hela cells treated by Bufalin. Moreover, we found that the expression of FAK, p-FAK (Tyr397), p-GSK3β (Ser389), AKT1 and p-AKT1(Ser473), which were downstream targets of integrins, were also decreased by Bufalin treatment, but the expression of GSK3β was enhanced. These results showed that integrin α2/β5/FAK signaling pathway played an important role in the anti-cancer effect of Bufalin.

To further confirm the role of integrin α2 in Bufalin-induced anti-cancer activities, we induced integrin α2 cDNA into Siha and Hela cells and subsequently treated these cells with Bufalin. As shown in Figure 3B, induction of integrin α2 cDNA could rescue restored the Bufalin-induced reduction of integrin α2 and β5 in the both cell lines. Correspondingly, Bufalin induced down-regulation of FAK, p-FAK (Tyr397), p-GSK3β (Ser389), AKT1 and p-AKT1(Ser473), was significantly abolished by over-expression of integrin α2 (Figure 3B), however, the expression of GSK3β was decreased due to the induction of integrin α2 cDNA (Figure 3B).

Next, we determined whether induction of integrin α2 cDNA could rescue the anti-cancer activities of Bufalin on the cervical cancer cells. Using a colony-formation assay, we found that the induction of integrin α2 cDNA drastically increased colony-forming ability of the cells upon exposure to Bufalin (Figure 3C and 3D). Furthermore, we found that up-regulation of integrin α2 significantly decreased Bufalin-mediated cell apoptosis (Figure 3E and 3F) and cell cycle arrest (Figure 3G and 3H). Besides, in transwell assay, cell invasion and migration suppressed by Bufalin was restored by integrin α2 cDNA introduction as shown in Figure 3I and 3J. In addition, we also found that, reduction of Bcl-2, cyclin A, MMP9 and Snail 1 caused by Bufalin was restored by introduction of integrin α2 cDNA, whereas, the increase of Bax, cyclin B1 and E-cadherin was suppressed by introduction of integrin α2 cDNA (Figure 3K). Taken together, our results demonstrated that Bufalin exhibited its anti-cancer activities through the inhibition of integrin α2/β5/FAK signal pathway.

### Bufalin sensitizes cervical cancer cells to paclitaxel treatment

**Figure 4:**
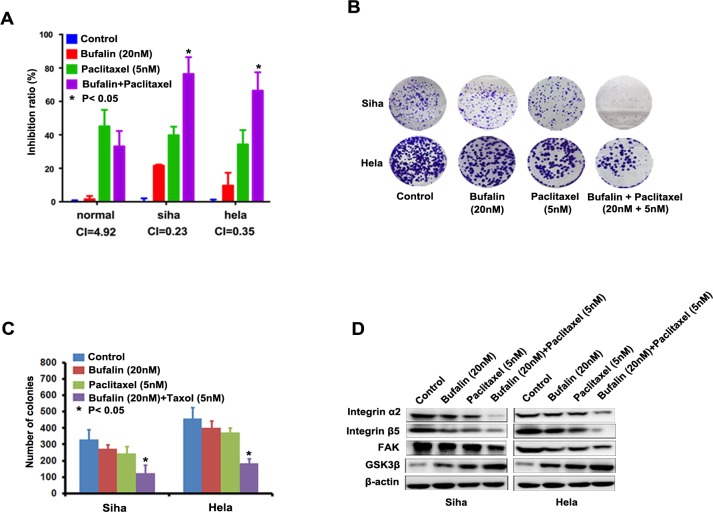
Bufalin sensitized cervical cancer cells to paclitaxel treatment **A.** The concurrent administration of cells with Bufalin and paclitaxel for 48 h results in synergistic inhibitory effect on the growth of Siha and Hela cells. B, Representative images and number of colonies formed by Siha and Hela cells treated with Bufalin or paclitaxel alone, and Bufalin plus paclitaxel. The experiments were repeated three times, and a representative experiment is shown. C, Western blot analysis showing that combination of RY-2f and cisplatin inhibited the lower expression of integrin α2, integrin β4, FAK and GSK3β than each agent alone.

Combinative chemotherapy is the main mode of clinical cancer therapy due to its significant advantages such as lower development of drug resistance and lower treatment failure rate. Platinum and paclitaxel currently is the firstline chemotherapeutic drug for cervical cancer in clinic. We detected the combined antitumor effect between platinum or paclitaxel and Bufalin in Hela and Siha cells. However, we found that Bufalin cannot enhance the anti-cancer effect of platinum paclitaxel in hela and siha cells (Supplemental Figure 1A and 1B). To check the combined effects of Bufalin and Paclitaxel on cervical cancer cells, cell survival was detected by CCK-8 assay. After combined treatment with Bufalin and Paclitaxel, the survival of cells was significantly reduced in compared with Bufalin or Paclitaxel alone treatment in both Siha and Hela cells, CI values of combination were lower than 1 (Figure 4A). Surprisingly, this combinative effect between Bufalin and Paclitaxel did not be observed in human normal cervical cells (Figure 4A). The result from colony-formation assay also showed that combined treatment with Bufalin and Paclitaxel decreased colony-forming ability of the cells upon exposure to Bufalin or Paclitaxel alone (Figure 4B and 4C). The combined treatment with Bufalin and Paclitaxel more efficiently inhibited the expression of integrin α2 /β5, FAK, GSK3β than Bufalin or Paclitaxel alone treatment in both Siha and Hela cells (Figure 4D). The above results showed that suggesting Bufalin, combined with paclitaxel, would be novel therapeutic agents for cervical cancer patients resistant to paclitaxel- based chemotherapy.

### Bufalin synergizes with Paclitaxel to inhibit the growth of cervical cancer *in vivo*

**Figure 5:**
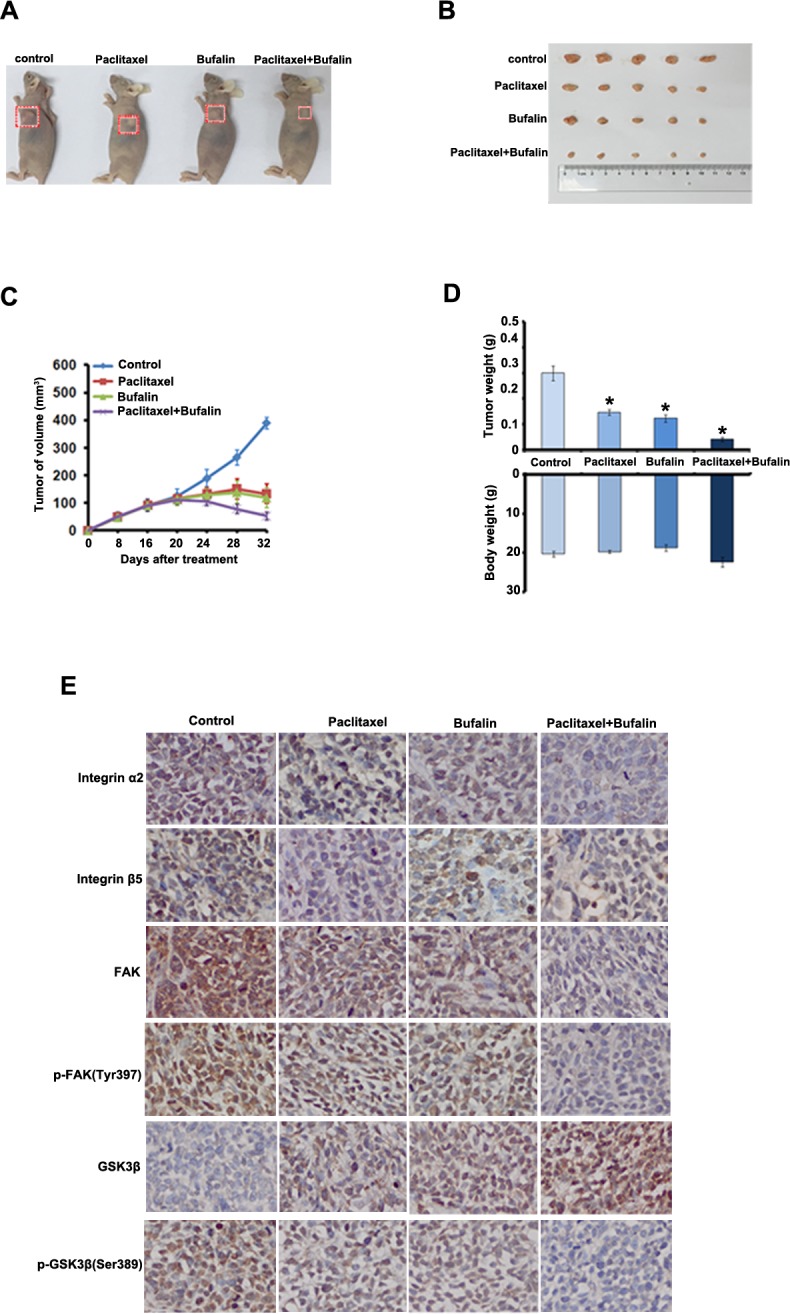
Inhibition of xenograft cervical cancer by Bufalin **A.-C.** Nude mice bearing tumors formed by Siha cells were administered with 10 mg/kg of Bufalin, 10 mg/kg of paclitaxel, and Bufalin (10 mg/kg) plus paclitaxel (10 mg/kg), respectively, or vehicle as controls (*n* = 5). Treatments were done given through intraperitoneal once every four days. Figures showed the average tumor volumes (A-C), red boxes showed the position of tumors in the nude mice. **D.** Figures showed tumor and body weights of mice at the end of observation. **E.** Immunohistochemical staining of in xenograft tumor tissues. Tissues were stained with rabbit anti-integrin α2, integrin β4, FAK, p-FAK(Tyr397), GSK3β and p- GSK3β (Ser389) antibodies and visualized with donkey anti-mouse secondary antibody (Magnification×200).

To test the synergistic antitumor effects of Bufalin and Paclitaxel *in vivo*, we generated the subcutaneous xenograft tumor models by transplanting Siha cells into nude mice. As shown in Figure 5A-5C, Bufalin (10 mg/kg per 4 days) or Paclitaxel (10 mg/kg per 4 days) treatment as single agent resulted in significant tumor volume reduction compared with treatment with dilent as control. In addition, compared with Bufalin or cisplatin alone treatment, co-treatment with Bufalin and Paclitaxel achieved further inhibition on the tumors growth by reducing the volume and weight of tumors (Figure 5D). The inhibition rates of tumor weight in the combined group were 75.2%, which were significantly higher than those in Bufalin (46.8%) or dinaciclib (41.3%) alone group (Figure 5D). More surprisingly, the body weights of mice in the combined group were slightly heavier than those in Bufalin or Paclitaxel alone treated group, suggesting co-treatment of Bufalin and Paclitaxel at the indicated dose caused certain side effects in mice (Figure 5D).

To determine whether the combination treatment could also resulted in decreasing of integrain/FAK signaling pathway *in vivo*, we performed IHC analyses on tumor sections from all the experimental groups. As shown in Figure 5E, compared with the single administration, combinative treatment further decreased the expression of integrin α2 and β5. Moreover, the expression levels of FAK, p-FAK(Tyr397) and p-GSK3β (Ser389) were largely reduced in the combination treatment group, but the expression of GSK3β was up-regulated. These results *in vivo* findings demonstrated that Bufalin exhibited anti-tumor activities and enhanced the anti-cancer efficacy of Paclitaxel through supressing integrin /FAK signaling pathway in cervical cancer.

## DISCUSSION

In this study, we fully described the anticancer role of Bufalin in cervical cancer cells, which provides an in depth understanding of cytotoxic mechanism of Bufalin in genetic level and offers more potentially useful biomarkers for diagnosis and treatment of cervical cancer in future.

The AKT/GSK3β pathway promotes cancer progression, including cellular proliferation, growth, survival, and drug resistance [28-32]. In the present study, we found that the expression level of AKT1, p-AKT1 (Ser473), and p-GSK3β (Ser389) were decreased in cervical cancer cells treated with Bufalin, but the expression of GSK3β was increased. Further, we found that the expressions of integrin α2, β5, FAK and p-FAK (Tyr397), the upstream regulatory proteins of AKT/GSK3β signal pathway, were simulated by the treatment of Bufalin. Meanwhile, induction of ITGA2 cDNA into cervical cancer cells could attenuate the anti-tumor effect of Bufalin and rescue the expression of FAK, p-FAK (Tyr397), AKT1, p-AKT1 (Ser473), GSK3β and p-GSK3β (Ser389), which indicated that Bufalin exerted its anti-tumor effects through the regulation of integrinα2β5/FAK/AKT1/ GSK3β signal cascade. Integrins mediate a wide variety of cell-cell and cell-matrix interactions that lead to cell migration, proliferation, differentiation and survival. For instance, integrin β1 interacted with CUB domain-containing protein-1 [33]and induced intracellular phosphorylation signaling, involving FAK and PI3K-dependent AKT activation to affect the metastasis of tumor cells. However, to our knowledge, it has not been reported that Bufalin exerts its anticancer effect through integrin/FAK signal pathway. Our results illustrated that Bufalin could inactivate AKT1 and activate the GSK3β by the suppression of the integrin/FAK signal pathway. These results showed that the cell membrane receptors and/or their coactivators might be new potential molecular targets for Bufalin in the cancer therapy.

Increasing studies have shown that bufalin could be used in combination with current clinical chemotherapeutic drug regimens such as gemcitabine and/or akt inhibitors to overcome drug resistance and improve the efficacy of treatments for patients with locally advanced cancers [11, 12]. Chen et al. found that the combination treatment with gemcitabine and Bufalin enhanced tumor cell growth inhibition compared with either agent alone in pancreatic cancer [12]. Zhu et al. reported that Bufalin synergized with Akt inhibitor LY294002 to induce the apoptosis of lung cancer A549 cells [11]. In our study, we also demonstrated that Bufalin synergized with paclitaxel to inhibit the proliferation and induce the apoptosis of cervical cancer cells at low concentrations, which had little damage to normal cervical cells. Based on the above studies, we suggest the combination with Bufalin and other clinical chemotherapeutic drugs will have a greater advantage in the treatment of the various types of cancers.

Recently, Calderon-Montano et al. proposed that the key feature of an efficient anticancer drug is its ability to kill (or inhibit the growth of) human cancer cells at concentrations that do not significantly affect human nonmalignant cells [34]. They further pointed out that it was not suitable to use rodent nonmalignant TM4 Sertoli cells to test the effect of Bufalin on nonmalignant cells [35]. Therefore, in this study, we cultured the human primary normal cervical cells from the patients in Fudan University Shanghai Cancer Center, and used these normal cervical cells to test the cytotoxicity of Bufalin. We found that Bufalin, at the lower concentration (< 20nM), had no apparent damage to normal cervical cells, but could inhibit the growth of cervical cancer cells, especially combined with paclitaxel. These results indicated Bufalin was a promising regimen for combined chemotherapy to overcome the resistance of various cancer cells.

In summary, we have demonstrated that Bufalin can induce cellular apoptosis and cell cycle arrest, reduce cell metastasis, and inhibit xenograft tumor growth mainly through repression of the integrin α2β5/FAK/ATK/GSK3β signal pathway. Therefore, Bufalin may be developed as a potential drug to treat human cervical cancer after further validation.

## MATERIALS AND METHODS

### Cell lines and cell culture

The established human cervical cancer cell lines SiHa and HeLa were obtained from American Type Culture Collection (ATCC). All cells were maintained in Dulbecco's modified Eagle's medium (DMEM, HyClone, Thermo Scientific, USA) supplemented with 10% fetal bovine serum (Gibco, Life technologies, USA), 100 U/ml penicillin (Biowest, Nuaillé, France), and 100 U/ml streptomycin (Biowest, Nuaillé, France) and incubated at 37°C in a humidified atmosphere with 5% CO_2_.

The primary human cervical cells were obtained from the cervical cancer patients at Fudan University Shanghai Cancer Center (FUSCC). The primary cells were maintained in Gibco^®^ Medium 199 (Gibco, Life technologies, USA), supplemented with 1μg/ml epidermal growth factor (EGF), 15% fetal bovine serum (Gibco, Life technologies, USA), 100 U/ml penicillin (Biowest, Nuaillé, France), and 100 U/ml streptomycin (Biowest, Nuaillé, France) and incubated at 37°C in a humidified atmosphere with 5% CO_2_.

### Drugs

Bufalin was purchased from Sigma-Aldrich (St Louis, MO) and paclitaxel was purchased from Bristol-Myers Squibb Company (NY, USA).

### Plasmids construction and viral infection

The recombinant plasmid pENTER-integrin α2 (ITGA2), containing human full cDNA sequence of ITGA2, was purchased from Vigene Biosciences (Jinan, China), and then the cDNA sequence of ITGA2 was subcloned into lentivirus vector pCDH-CMV-MCS-EF1-PURO, generating the recombitant plasmid pCDH/ITGA2 cDNA. Lentivirus carrying ITGA2 cDNA were generated and harvested as described previously. Briefly, Siha and Hela cells were infected twice for a total of 4 days (2 days for each infection) and the positive clones were selected with puromycin (200ng/mL) for 7-10days. Control cell lines were generated by infection with viruses containing the empty vector by following the same protocol.

### *In vitro* cytotoxicity

The *in vitro* cytotoxicity of Bufalin was measured by Cell Counting Kit-8(CCK-8) (Dojindo Laboratories, Kumamoto, Japan), as described in the literature. Briefly, 5×10^3^ cells per well were plated in 96-well plates and treated with Bufalin, paclitaxel, Bufalin plus paclitaxel or DMSO (diluent) at various concentrations for 48 h. Then, the medium with compounds or DMSO was replaced with 180 μL of fresh medium along with 20 μL CCK-8 solution in each well and incubated at 37°C for 2 h. Last, the CCK-8-containing medium was discarded and 150 μL of DMSO per well was added to dissolve the newly formed formazan crystals. Absorbance of each well was determined by a microplate reader (Synergy H4, Bio-Tek) at a 450 nm wavelength. Growth inhibition rates were calculated with the following equation, Inhibition ratio = (OD_DMSO_-OD_drug_)/(OD_DMSO_-OD_blank_)×100%.

### Colony formation assay

1×10^3^ cells were seeded in six-well plates at a single cell density and treated with Bufalin, paclitaxel, Bufalin plus paclitaxel or DMSO (diluent) at various concentrations for 24h, 48 h and 72h. Then the fresh medium was added to allow cell growth for at least one week. The colonies with more than 50 cells were counted after staining with gentian violet (Solarbio).

### Cell cycle analysis

Cell cycle status was detected by flow cytometry according to a previously published method [26] and analyzed by Multicycle AV (for windows, version 320) software. Briefly, cells were first treated with RY-2f or DMSO at various concentrations for 48 h, and then harvested, washed twice with 1× PBS, and re-suspended in 200 μL of 1× PBS. The cells were fixed in 4 mL of ice-cold 75% ethanol at 4°C overnight and stained with 200 μL of propidium iodide (50 μg/mL, Sigma-Aldrich) and 20 μL of RNase (1 mg/mL, Sigma-Aldrich) to remove RNA in a 37°C water bath for 15 to 20 minutes. The cells were then analyzed by flow cytometry (Cytomics FC 500 MPL, Beckman Coulter). The results were indicated as mean values from three independent determinations.

### Cell apoptosis analysis

To detect apoptosis, cells were incubated with Bufalin or DMSO at different concentrations for 48 h. The cells were harvested, washed twice with cold 1×PBS, and re-suspended in 200 μL binding buffer at density of 1 × 10^5^ cells / mL. The cells were then stained with 5 μL Annexin-V and PI (BD Biosciences) for 15 min in dark condition at room temperature and subjected to analysis by flow cytometry (Cytomics FC 500 MPL, Beckman Coulter). The early apoptosis was evaluated based on the percentage of cells with Annexin V+/PI-, while the late apoptosis was that of cells with Annexin V+/PI+. The results were indicated as mean values from three independent determinations.

### Cell invasion and migration assay

A high throughput screening multi-well insert 24-well two-chamber plate (BD Biosciences, San Jose, CA), with an 8-μm (pore size) polycarbonate filter between chambers were used to test cell invasion and migration. To test cell invasion, 2.5 × 10^3^ cells of Siha or Hela treated with Bufalin at different concentrations and their corresponding controls were added in upper chamber and allowed to invade at 37°C for 24 hours toward a lower reservoir containing medium plus fibronectin (20μg/mL). To examine cell migration, 3 × 10^4^ cells of Siha or Hela treated with Bufalin at different concentrations and their corresponding controls were added in upper chamber and allowed to invade at 37°C for 24 hours toward a lower reservoir containing medium without fibronectin. The cells were then fixed in 100% methanol for 30 minutes and stained with Giemsa solution for 10 minutes. The invasive cells were counted as those passed through the membrane separating the chamber. All cells were counted at ×200 magnification under a microscope. The assay was repeated three times with duplicate. Scratch assay was also used to examine cell migration and cells were incubated in 6-well plate over-night to yield monolayer confluence for scratch assay. Scratches were made using a pipette tip and photographed immediately (time 0) and 24 hours’ later. The distance migrated by the cell monolayer to close the scratch area during the time period was measured. Results were analyzed as migration index, which was the ratio of the cell migration distance at 24h to that at 0h. The assay was carried out in triplicate and repeated three times.

### Western blot analysis

Western blot analysis was performed to determine the expression levels of various proteins in cells. Cells were treated with Bufalin or DMSO at different concentrations for 48 h. Cells were harvested, washed with cold 1×PBS, and lysed with RIPA lysis buffer (Beyotime) for 30 min on ice, then centrifuged at 12,000 *g* for 15 min at 4°C. The total protein concentration was determined by BCA protein assay kit (Beyotime). Equal amounts (30 μg per load) of protein samples were subjected to SDS-PAGE electrophoresis and transferred on to polyvinylidene fluoride (PVDF) membranes (Millipore). The blots were blocked in 10% non-fat milk, and incubated with primary antibodies, followed by incubation with secondary antibodies conjugated with horseradish peroxidase (HRP). The protein bands were developed with the chemiluminescent reagents (Millipore). Antibodies to p21^waf/cip1^, p27^cip/kip^, Bcl-2, Bax, Bcl-xl, cyclin A, cyclin B1, CDK2, MMP9, E-cadherin, Snail1, pAKT(Ser437) and AKT1 were from Santa Cruz Biotechnology. Antibodies to integrin α2 (ITGA2), integrin β5 (ITGB5), FAK, p-FAK (Tyr397), GSK3β and p- GSK3β (Ser389) were from Cell Signaling Technology. The antibody to β-Actin was purchased from Sigma-Aldrich.

### Immunofluorescence

Immunofluorescence staining was done according to a published protocol [27]. Primary antibodies against Snail1 were obtained from Santa Cruz Technology (California, US). DNA dye 4′,6-diamidino-2-phenylindole (DAPI) was obtained from Molecular Probes. The secondary antibodies used were the cy3-conjugated donkey anti-rabbit IgG (Jackson ImmunoResearch Laboratory). All stained cells were examined and photographed with a Leica SP5 confocal fluorescence microscope.

### *In vivo* tumor growth assay

Female BALB/c nude mice at 4-5 weeks of age were purchased from Shanghai Slac Laboratory Animal Co. Ltd. and housed in a specific pathogen free facility. Mice were subcutaneously inoculated with Siha cells (3×10^6^ suspended in 0.2 mL PBS for each mouse). After reaching an average tumor volume of 100 mm^3^, the animals were randomized into groups (*n* = 5) that were treated intraperitoneally with either Bufalin (10 mg/kg), paclitaxel (5 mg/kg), Bufalin (10 mg/kg) plus paclitaxel (5 mg/kg), or vehicle control (0.2 mL olive oil) thereafter. Administration of vehicle or agents and measurement of tumor growth with a digital caliper were done once every 4 days. Tumor volumes were calculated by the two dimensional sizes of each tumor with the following formula: V = L × W^2^ ×0.52, where V is the volume, L is the length, and W is the width. At the end of experiment, the mice were weighed and sacrificed, and the tumors were weighed and dissected. The animal experimental protocols were approved by the Animal Ethics Committee of Fudan University Shanghai Cancer Center.

### Statistical analysis

The data in this study were calculated using Graph Pad Prism and expressed as mean ± S.E. A nonlinear regression model with a sigmoidal dose response was used to fit the values of IC50. Comparisons between controls and treated groups were determined by paired *t* test or one-way ANOVA followed by Tukey's multiple comparison tests. All results were considered statistically significant at the level of *p <* 0.05 and combination index was calculated by CompuSyn software.

## SUPPLEMENTARY MATERIAL FIGURE


