# Supplementary material for: Bufalin enhances antitumor effect of paclitaxel on cervical tumorigenesis via inhibiting the integrin α2/β5/FAK signaling pathway
Source: Oncotarget. 2016 Jan 7;7(8):8896–907. doi: 10.18632/oncotarget.6840 (PMC4891012; doi:10.18632/oncotarget.6840)
Supplement: Supplementary file 1 [file oncotarget-07-8896-s001.pdf]

Bufalin enhances antitumor effect of paclitaxel on cervical tumorigenesis *via* inhibiting the integrin α2/β5/FAK signaling pathway

Supplementary Material

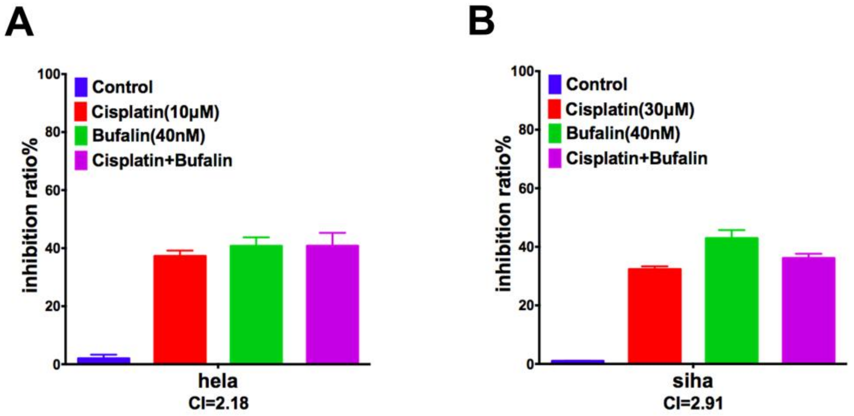

Supplemental Figure 1. Combination of Bufalin and platinum had no synergistic effect on cervical cancer cells

A-B, The concurrent administration of cells with Bufalin and platinum for 48 h did not result in synergistic inhibitory effect on the growth of Hela and Siha cells.
